# A cell-based assay for CD63-containing extracellular vesicles

**DOI:** 10.1371/journal.pone.0220007

**Published:** 2019-07-24

**Authors:** Anil G. Cashikar, Phyllis I. Hanson

**Affiliations:** Department of Cell Biology & Physiology, Washington University School of Medicine, St. Louis, Missouri, United States of America; Institut Curie, FRANCE

## Abstract

Extracellular vesicles (EVs) are thought to be important in cell-cell communication and have elicited extraordinary interest as potential biomarkers of disease. However, quantitative methods to enable elucidation of mechanisms underlying release are few. Here, we describe a cell-based assay for monitoring EV release using the EV-enriched tetraspanin CD63 fused to the small, ATP-independent reporter enzyme, Nanoluciferase. Release of CD63-containing EVs from stably expressing cell lines was monitored by comparing luciferase activity in culture media to that remaining in cells. HEK293, U2OS, U87 and SKMel28 cells released 0.3%-0.6% of total cellular CD63 in the form of EVs over 5 hrs, varying by cell line. To identify cellular machinery important for secretion of CD63-containing EVs, we performed a screen of biologically active chemicals in HEK293 cells. While a majority of compounds did not significantly affect EV release, treating cells with the plecomacrolides bafilomycin or concanamycin, known to inhibit the V-ATPase, dramatically increased EV release. Interestingly, alkalization of the endosomal lumen using weak bases had no effect, suggesting a pH-independent enhancement of EV release by V-ATPase inhibitors. The ability to quantify EVs in small samples will enable future detailed studies of release kinetics as well as further chemical and genetic screening to define pathways involved in EV secretion.

## Introduction

Extracellular vesicles (EVs) are released by cells and found in most biological fluids including urine, plasma, cerebrospinal fluid, saliva etc. as well as in tissue culture conditioned media. EVs are thought to mediate cell-cell communication [1] and may carry a variety of proteins, lipids and RNA with potential to impact target cell physiology. It has been proposed that EVs modulate tumor environments to allow for tumor seeding and growth and promote angiogenesis [2–8]. EVs have also been implicated in the prion-like spread of neuropathogenic protein aggregates in several neurodegenerative diseases [9–15]. Certain viruses and bacteria such as hepatitis A virus [16], herpesvirus 6 [17], HTLV-1 [18], HIV [19,20] and uropathogenic *E*. *coli* [21,22] may use the cellular pathways of EV biogenesis for extracellular release. Recent studies in many laboratories have focused on exploring the utility of EVs isolated directly from biological fluids as disease biomarkers [23,24]. Finally, EVs are also being developed as therapeutic agents capable of delivering drugs to specific tissues or organs in the body [3,25,26].

EVs are produced in at least two distinct ways. Fusion of endosomal multivesicular bodies (MVBs) with the plasma membrane releases intraluminal vesicles in the form of exosomes whereas direct outward budding of the plasma membrane generates ectosomes, also referred to as microvesicles. Based on the origin of EVs a number of proteins are well established as markers. Among these are the tetraspanins characterized by four membrane spanning helices, including most notably CD63 [27–31]. CD63 is predominantly localized to the intraluminal vesicles (ILVs) of late endosomes and MVBs and is thus enriched on exosomes [31–33]. Cytoplasmic proteins including members of the endosomal sorting complex required for transport (ESCRT) machinery, syntenin, and certain chaperones are also enriched in EVs [32,34]. In addition, a variety of RNAs are present and thought to carry signals involved in cell-cell communication [35–37]. Several packaged miRNAs have been shown to exert specific biological effects supporting the notion that EV cargo is actively selected. Mechanistic understanding of how cargo is selected and packaged into EVs and how EV release is regulated will be important for future progress in this area.

EVs are small, ranging in size from ~50-140nm in diameter, making it difficult to monitor them using light-based methods [38]. Most studies of EVs depend on efficiently collecting them from cell culture media or biological fluids using high speed centrifugation or precipitation-based methods [39]. As cultured cells produce small quantities of EVs, analytical studies tend to require large volumes of conditioned media from cells cultured over one or more days which may be contaminated with material associated with cell injury and death. Moreover, typically used isolation procedures are inefficient, with extensive losses that preclude reproducible and quantitative yield [40]. Thus, better methods are needed to quantify EVs in small samples to facilitate chemical and genetic screens both to elucidate cell biological mechanisms involved in EV biogenesis and to identify strategies to modulate EV production.

Here, we have developed a new cell-based assay system to quantitate EV release with high sensitivity using stable cell lines expressing CD63 fused to a nanoluciferase reporter. Using this assay system, we screen a set of known biologically active compounds to reveal pathway(s) impacting EV release.

## Materials and methods

### Cells and Reagents

T-REx-293 cells were from Thermo Fisher (Cat# R71007). U2-OS, U87-MG and SKMel28 were purchased from the American Type Culture Collection (Manassas, VA). Cells were transfected with the appropriate plasmids and stable transfectants were selected using 100 μg/ml Zeocin (Thermo Cat #R25005) in DMEM (Gibco) with 10% fetal bovine serum (Atlanta Biologicals). Nano-Glo Luciferase Assay System was purchased from Promega (Cat # N1110). Antibodies used in this study are listed in S1 Table.

### Plasmid construction

Plasmid pNL1.1[*Nluc*] carrying Nanoluciferase cDNA was a gift from Promega (Cat# N1001). The Nluc gene was amplified using PCR with an upstream HA-tag along with appropriate cloning sites for expression as either a soluble cytoplasmic protein (HANL) or as fusion at the N-terminal end of CD63 gene (HANLCD63) in pcDNA4/TO plasmid (Thermo Fisher, Cat# V102020) backbone.

### Nanoluciferase assays

TREx293 cells expressing HANL or HANLCD63 were grown in media without tetracycline and induced with 1μg/ml tetracycline overnight (17h). We also compared this with cells continuously maintained in media containing a low concentration of tetracycline (0.2μg/ml) and found no differences between the two paradigms. In this study, we maintained cells in media containing 0.2μg/ml Tet to maintain cells in a constitutively ‘on’ state. Typically, 50–100,000 cells per well were plated in a 24-well plate as needed for the conditions to be tested. After 18-24h for cell attachment, cells were washed twice with media to remove previously released EVs. Fresh media containing appropriate drugs (as indicated in the figures) was added to cells in 0.5ml media per well. Effects of the drugs were measured after indicated duration (typically 5 hours). At the end of the treatment, 250μl of conditioned media was removed into labeled 1.5ml tubes and centrifuged at 16000g for 5 minutes to remove large cell debris. Supernatants (media & EV) were collected into fresh tubes. To the cells in the remaining 250μl of media in the 24-well plate, 5μl of 10% Triton X100 was added (final 0.2%) to permeabilize cells and to release all contents (total). We confirmed that Triton X100 does not alter Nluc activity in HANL or HANLCD63 samples (see S2 Fig). 50μl of ‘media & EV’ samples and 1:10 diluted ‘total’ samples (5μl of sample+45ul of blank media) were transferred to 96-well plates (Costar #3915). To each well containing sample, 25μl of Nano-Glo luciferase assay (Promega Cat# N1110) reagent (prepared as per manufacturer’s instructions) was added. Luminescence was measured immediately after reagent addition using a microplate reader (BioTek Synergy hybrid H2). Background luminescence in media-only blanks was also measured within each plate. Samples were measured in duplicates or triplicates and averages were plotted. To obtain an unbiased quantitation of EVs in various samples, a ratio of luminescence in ‘media & EV’ to that in ‘total’ cell lystates is reported, termed ‘Fractional Release’.

### Isolation of EVs by PEG precipitation

For precipitation of EVs from conditioned media, 1/3^rd^ volume of 40% PEG 3350 prepared in PBS was added to conditioned media for a final concentration of 10% PEG and incubated at 4C overnight. The mixture was centrifuged at 16,000g for 30 minutes at 4C. The pellet was resuspended in PBS.

### Immunofluorescence

For immunofluorescence experiments, 50,000 cells were plated in 0.5ml on untreated #1.5 coverslips (12mm circle) in separate wells of a 24-well plate. Cells were fixed in 4% paraformaldehyde in PBS for 20 minutes at room temperature and moved to fresh wells containing 1ml of PBS. Fixed cells were blocked and permeabilized in PBS containing 10% goat serum and 0.2% saponin for 1h. Blocked coverslips were incubated in primary antibody dilutions made in the blocking solution followed by 3 washes in PBS. Coverslips were incubated in secondary antibody dilutions made in the blocking solution followed by 3 washes in PBS. The coverslips were given a final rinse in PBS, mounted on clean glass slides with gelvatol and allowed to set overnight. Images were collected using a Nikon spinning disk confocal microscope equipped with a CCD camera and Nikon Elements software.

### Immunoblotting

For immunoblotting experiments, cells were seeded at a density of 200,000 cells per well of a 6-well plate with 3ml of growth medium. The conditioned media was collected and subjected to PEG precipitation as described above. Cells were collected in 1ml of PBS into a 1.5ml tube and pelleted at 1000g for 3 minutes. The cell pellet was lysed in 100μl of hypotonic lysis buffer containing 50mM Tris.HCl (pH 7.5), 0.1% sodium dodecyl sulfate (SDS), 1X protease inhibitor cocktail (Roche) and 250 units/ml Benzonase (Sigma). After 5 minutes at room temperature, 35μl of 4X SDS-PAGE loading dye with 2-mercaptoethanol (for anti-HA blots) or without 2-mercaptoethanol (for anti-CD63 blots) was added and the samples were boiled for 5 minutes. Samples were separated by SDS-PAGE and blotted onto nitrocellulose membrane. Membrane was blocked with 10% non-fat dry milk in TBS with 0.1% Tween 20, followed by incubations in primary and secondary antibody dilutions with 3 washes after each step. Blots were developed using chemiluminescence and imaged on a BioRad Chemidoc. Bands were quantified using the BioRad ImageLab software.

## Results

### Model development

**Fig 1:**
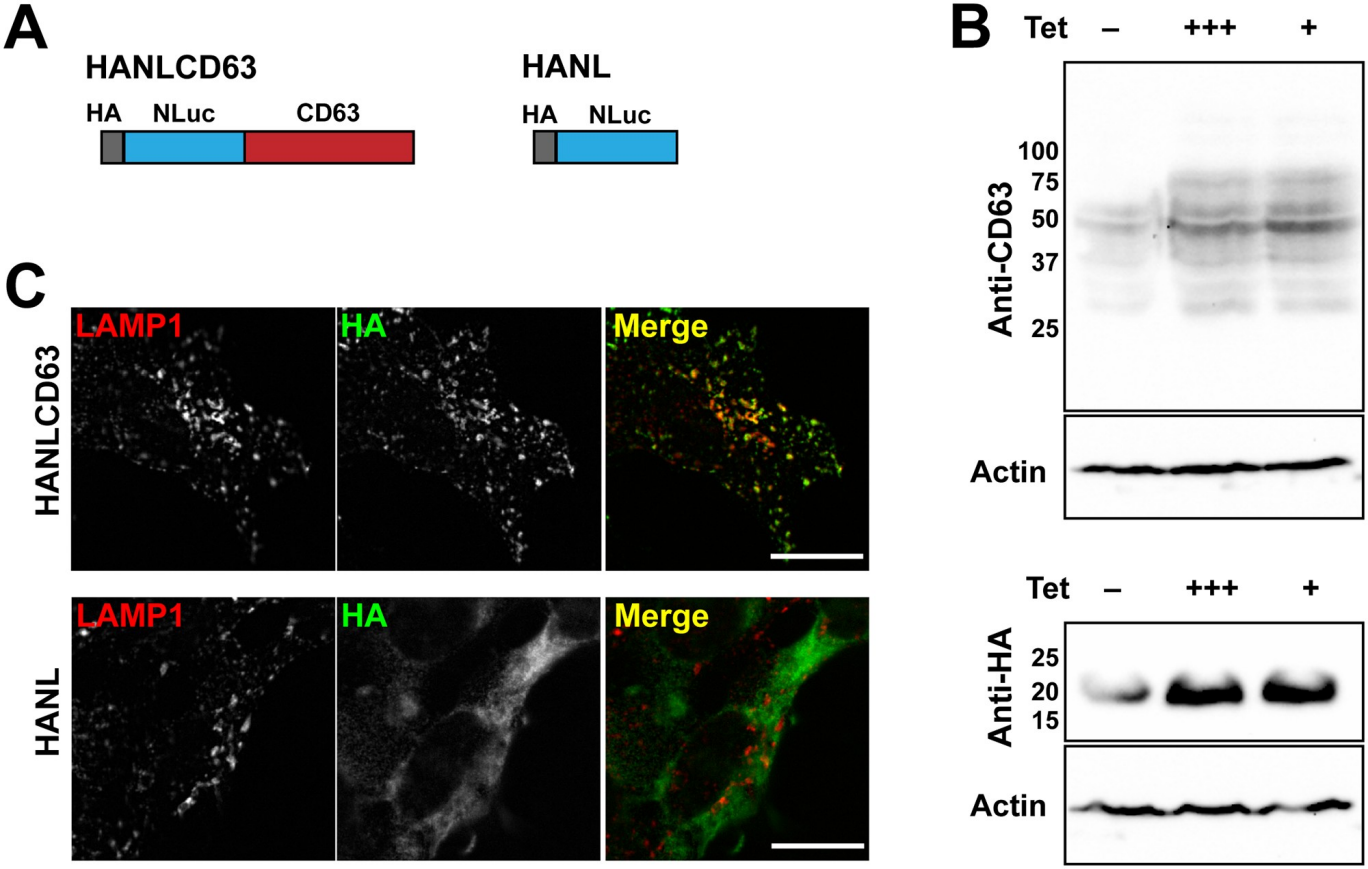
Development of a model system to quantify CD63-containing EVs. (A) Schematic diagram showing the locations of HA-tag, Nanoluciferase and CD63. (B) Immunoblots of lysates of stably transfected TRex293 cells show inducible expression of HANLCD63 (top, anti-CD63 blot) and HANL (bottom, anti-HA blot). Samples from cells grown in the absence of Tet (-), in the presence of high Tet (+++, 1μg/ml for 17h), or when continuously cultured in low Tet (+, 0.2μg/ml over 3 passages). Lysates were blotted for actin to confirm equal loading. (C) Immunofluorescence of stably transfected TRex293 cells cultured in media containing low concentrations of Tet to express HANLCD63 or HANL were immunostained for LAMP1 and HA epitope tag. Bar = 10μm.

To develop a high sensitivity assay for detecting CD63-containing EVs released by limited numbers of cells over a short time into small volumes of culture media, we fused CD63 to the smallest available reporter luciferase known as Nanoluciferase (Nluc). In addition to fusing Nluc to CD63, we also developed a soluble Nluc reporter to monitor non-specific leakage of cellular contents resulting from cell injury or death. Nluc is a 19kDa luciferase based on a naturally occurring monomeric luciferase from the deep-sea shrimp, *Oplophorus sp*. [41]. Nluc utilizes the coelenterazine analog furimazine as substrate to produce a bright glow-type luminescence in an ATP-independent reaction. Nluc has been engineered for use in a variety of applications requiring quantitative and sensitive measurements [41]. To enable antibody-based monitoring of the fusion proteins we introduced a hemagglutinin (HA) tag at the N-terminus of Nluc to generate HANL (Fig 1A). Since CD63 has a GYXXΦ-motif near its C-terminus that binds clathrin adaptor proteins and is involved in sorting [42] and GFP fusions at the N-terminus have been successful in previous studies [43,44], we fused HANL to the cytoplasmic N-terminus of CD63 to generate HANLCD63 (Fig 1A). HANL and HANLCD63 were cloned under a tetracycline (Tet)-inducible promoter and transfected into cells for selection of stable cell lines.

First, we examined the expression of these fusion proteins in Tet-inducible human embryonic kidney cells, TRex293 (HEK293), by immunoblotting (Fig 1B). Expression of HANL and HANLCD63 was induced by adding Tet to TRex293 cell lines. We compared expression in cells incubated overnight with a high concentration (1μg/ml) of Tet to that in cells grown in the continuous presence of a low concentration (0.2μg/ml) of Tet over three passages (about 10 generations). Tet-induced expression of HANL or HANLCD63 did not affect the rate of cell growth or cell number relative to uninduced controls (data not shown). Immunoblotting cell lysates for CD63 revealed that HANLCD63 was expressed inducibly at a level comparable to or less than that of endogenous CD63 (Fig 1B). Immunoblotting HANL samples for HA showed that HANL was strongly induced in response to Tet (Fig 1B). Quantitation of bands in Fig 1B showed that in Tet-induced samples expressing HANLCD63, an additional band at ~75kDa (band#1 in S1 Fig) corresponding to HANLCD63 was observed. Relative quantitation of endogenous CD63 and HANLCD63 was not feasible since both endogenous CD63 and HANLCD63 migrated as multiple bands (Fig 1B and S1 Fig). Similar results were obtained using Tet-inducible U2OS cells (S1 Fig).

Next, we examined HANLCD63 and HANL localization by immunofluorescence and confocal microscopy (Fig 1C). We compared the distribution of HA-tag with that of the late endosome/lysosome protein, LAMP1. In cells expressing HANLCD63, HA-signal was distributed similarly to that of LAMP1 on intracellular organelles. A small proportion of HANLCD63 was also observed on the plasma membrane as expected for endogenous and overexpressed CD63 [44]. In contrast, HA staining in HANL expressing cells was diffusely present throughout the cytoplasm and distinct from LAMP1 confirming its cytoplasmic distribution as expected. HANLCD63 expressed in U2OS cells also mostly colocalized with LAMP1 (S1 Fig).

### EV release

**Fig 2:**
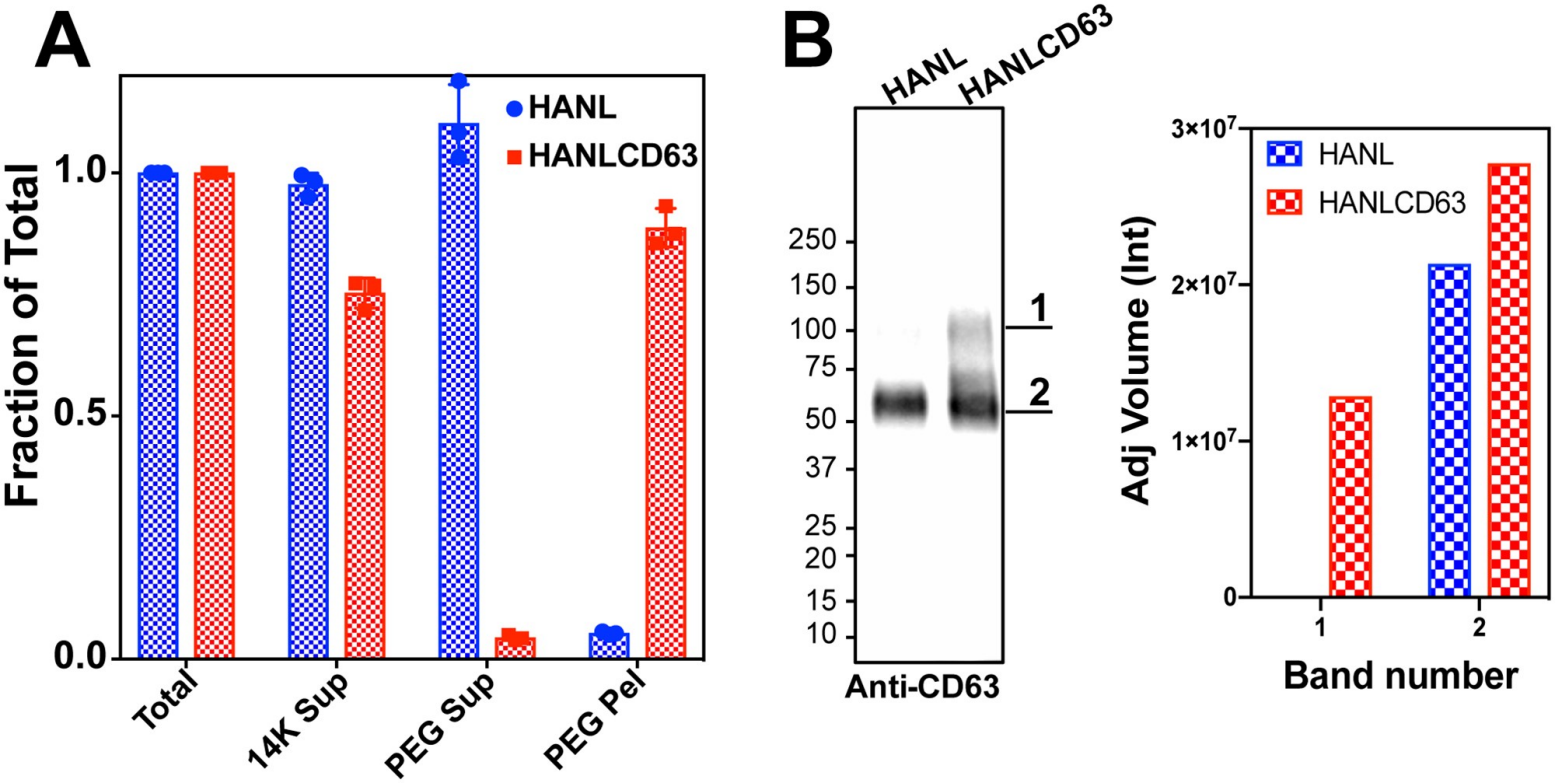
Characterization of EV release using HANLCD63. (A) Precipitation of EVs using polyethylene glycol (PEG). Samples are–complete conditioned media (Total); supernatant after centrifugation at 14000g (14K sup); supernatant after PEG precipitation (PEG sup); pellet after PEG precipitation (PEG pel). (B) PEG pellets immunoblotted for CD63. Band intensities were quantified using the BioRad ImageLab software.

To determine whether HANLCD63 will be useful as a marker of EV release, we next tested if released HANLCD63 is detectable in conditioned media in association with EVs. To do this, we added 10% polyethylene glycol (PEG) to conditioned cell media as described earlier [44] and measured Nluc activity in PEG soluble and insoluble fractions (Fig 2A). Although PEG precipitation is not selective for EVs, this method provides a simple and robust method for quantitative collection of EV-associated CD63 from conditioned media [44]. In HANL samples nearly all of the Nluc activity was found in the PEG soluble fraction. In HANLCD63 samples most of the Nluc activity was found in the PEG insoluble fraction consistent with the presence of HANLCD63 in EVs. We further confirmed this by immunoblotting PEG pellets with CD63 antibody (Fig 2B). In PEG pellets from HANL cell media only a single band of endogenous CD63 was detected with molecular weight of 50-60kDa (band# 2), whereas in HANLCD63 derived samples additional bands at higher molecular weights (band# 1) were observed consistent with additional recovery of HA tagged HANLCD63. The proportional signal in bands associated with HANLCD63 and endogenous CD63 is similar in cell lysates (shown in Fig 1B) and EVs (shown in Fig 2B), suggesting that the tagged and endogenous protein are incorporated similarly into EVs. Together with the localization of HANLCD63 to LAMP1-marked late endosomes and lysosomes, we conclude that the Nluc activity of HANLCD63 can be used to monitor EV associated CD63.

**Fig 3:**
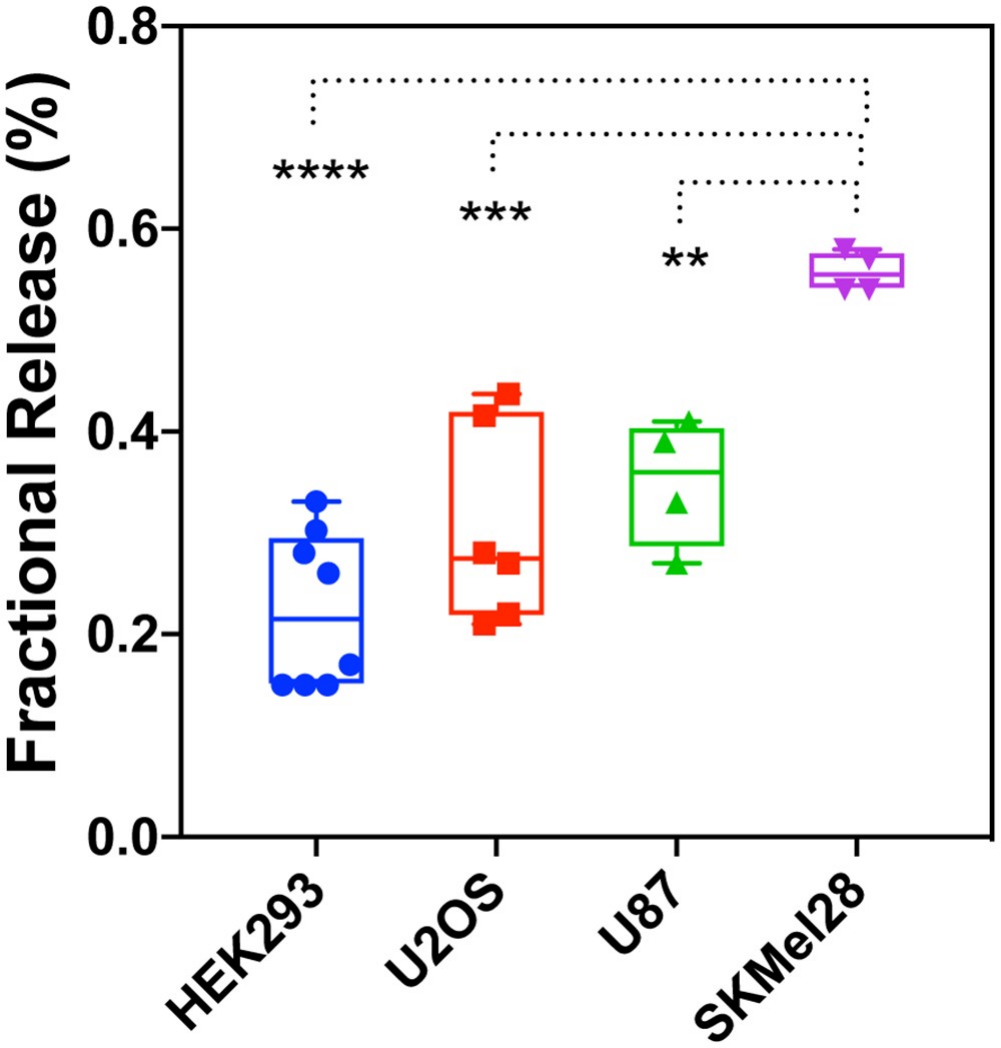
EV release in different cell lines. Comparison of EV release from TRex293 (HEK293), U2OS, U87 and SKMel28 cells. Fractional release was compared using ordinary one-way ANOVA with Tukey’s multiple comparisons test. ** = 0.005; *** = 0.0003; **** <0.0001.

To quantitatively track EV release from HANLCD63 expressing cells, we measured Nluc activity in conditioned media as well as the total Nluc activity in HANLCD63 expressing cells. This allowed us to report EV release in comparative terms as the ratio of extracellular to total Nluc activity or ‘fractional release’. To do this, we collected conditioned media at the time indicated and then replaced it with same volume of media containing 0.2% Triton X100 to extract HANLCD63 remaining in the cells. (See S2 Fig showing no effect of Triton X100 on Nluc activity of HANL and HANLCD63). In TRex293 (HEK293) cells, a small portion (0.1–0.3%) of total cellular HANLCD63 was released in EVs during a 5 hr collection period (Fig 3). For insight into how fractional release of EVs and cytoplasmic proteins varies among different cell lines, we generated and characterized U2OS, SKMel28 and U87-MG cells stably expressing HANLCD63. HANLCD63 was localized to LAMP1-positive endosomes as in TRex293 cells (Fig 1C, S1 Fig and data not shown). Interestingly, different cell lines exhibited different levels of EV release over a 5h period (Fig 3). While HEK293, U2OS and U87 cells showed a fractional release of between 0.1%-0.4%, SKMel28 cells released about 0.6% of total cellular HANLCD63. This result underscores the utility of this assay system for defining EV production by different types of cells. We used stably transfected TRex293 cells for most experiments in this study.

**Fig 4:**
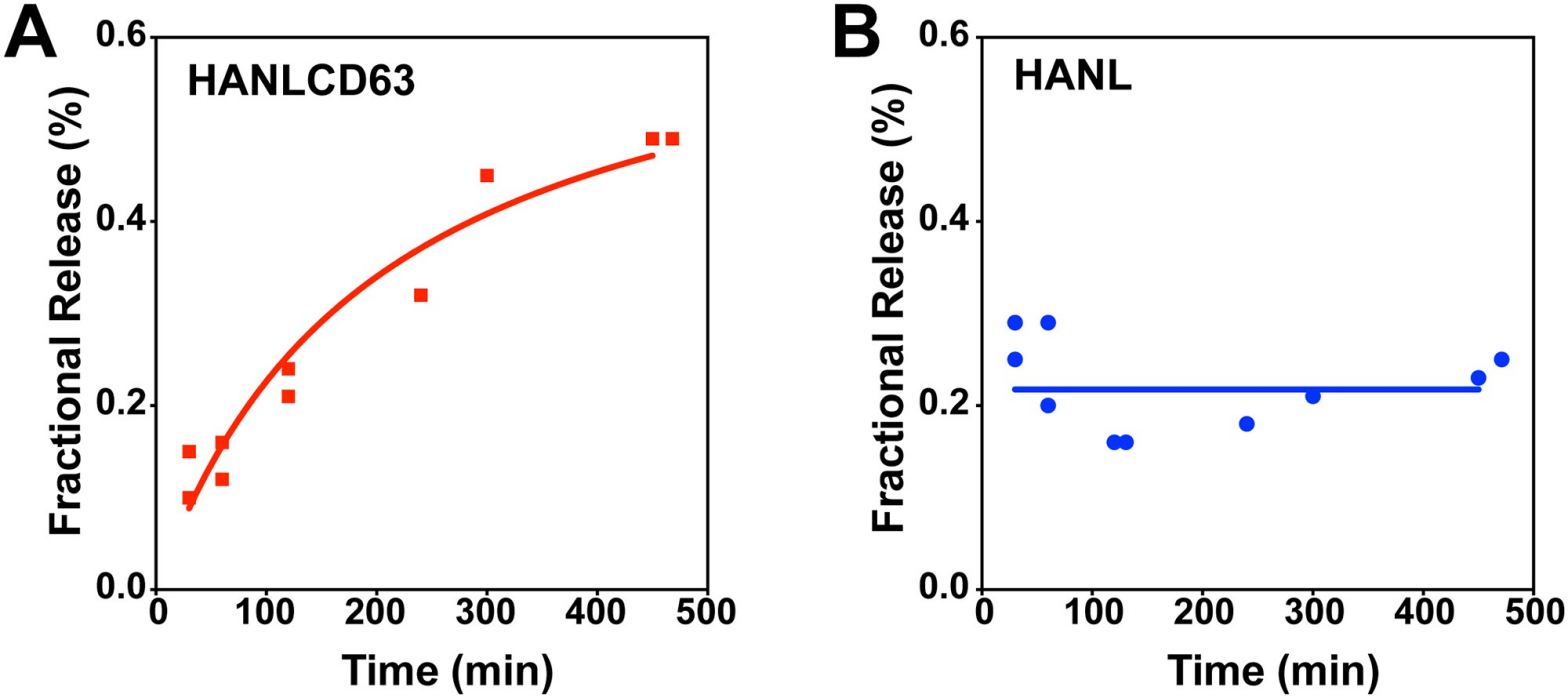
Kinetics of EV release. EV release over 6h as measured with HANLCD63 (A). (B) Extracellular release of cytoplasmic HANL protein over 6h.

Important clues to understanding EV biogenesis and how it can be modulated can come from monitoring the kinetics of EV release. Given the inefficiencies of current exosome isolation methods, EV release has, however, mostly been studied after single prolonged collection times without time resolution or specific normalization to parent cells. Our model system is well suited to monitoring the kinetics of EV release. To measure fractional release of EV associated HANLCD63 or cytoplasmic HANL at different times after initiating collection, conditioned media and cell extracts were collected and used to measure Nluc activity. The time course of HANLCD63 release shows that low initial amounts of Nluc activity in the media (roughly 0.1% of cellular HANLCD63) steadily increase over 6h, reaching a fractional release of approximately 0.5%. This five-fold increase demonstrates both the sensitivity of this model system for detecting changes in EV release and, importantly, that under these baseline conditions EVs are being continuously released. In cells expressing HANL, the amount of Nluc present in the media (roughly 0.2–0.3% of cellular HANL) remains unchanged over 6 hours (Fig 4A & 4B). Based on the fact that most extracellular HANL was not precipitated by adding PEG (Fig 2A), we interpret its presence to reflect some form of cell or membrane damage, most likely associated with media change and washing at the beginning of the experiment.

### Screen for pathway perturbants

**Fig 5:**
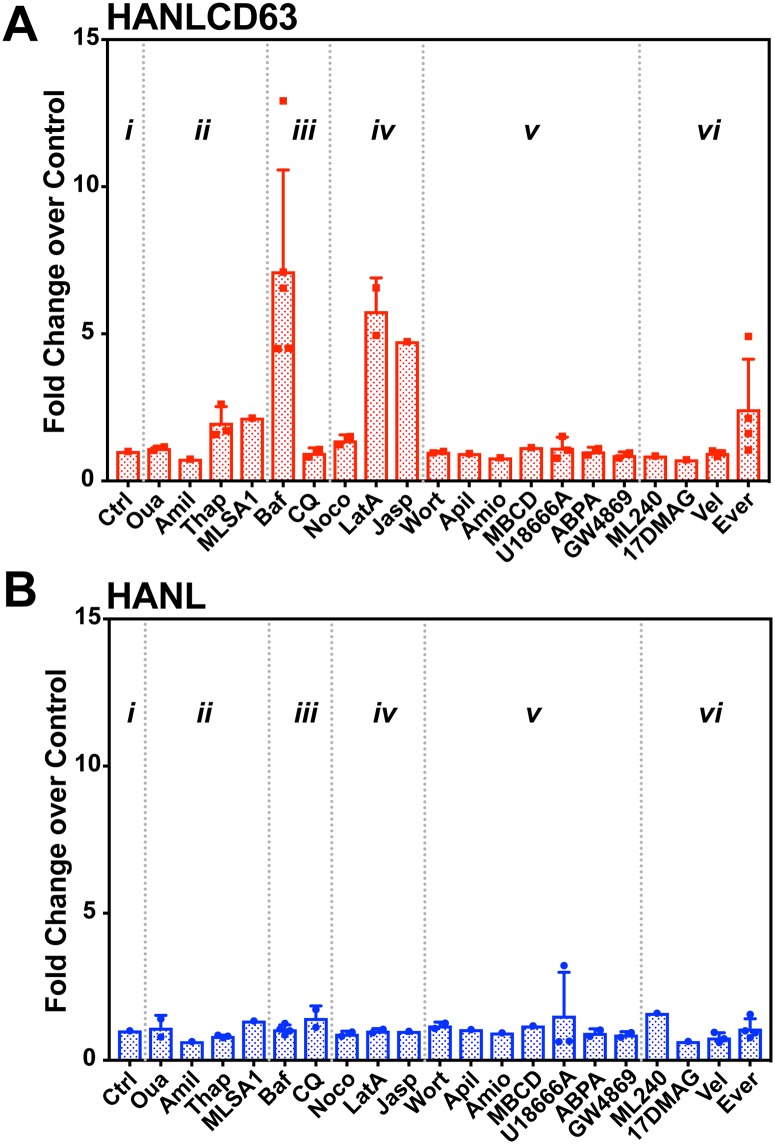
Screen for pathway perturbants. HANLCD63 (A) and HANL (B) expressing TRex293 cells were treated with indicated compounds during a 5h EV collection. Nluc activity in media is shown as fold change over that released by control cells (Ctrl). Compounds were used at the following concentrations: (i) Control—Ctrl (No drugs); (ii) Ion balance perturbants—Oua (175nM Ouabain); Amil (100nM Amiloride); Thap (1μM Thapsigargin); MLSA1 (50μM MLSA1); (iii) Lysosome perturbants—Baf (200nM Bafilomycin); CQ (50μM Chloroquine); (iv) Cytoskeleton perturbants—Noco (1uμg/ml Nocodazole); LatA (2μM LatrunculinA); Jasp (2μM Jasplakinolide); (v) Lipid perturbants—Wort (10μM Wortmannin); Apil (2μM Apilimod); Amio (10μM Amiodarone); MBCD (1mM MβCyclodextrin); U18666A (7μM U18666A); ABPA (2μM 1-aminodecylidene bis-phosphonic acid); GW4869 (17.3μM GW4869); (vi) Other perturbants—ML240 (5μM ML240); 17DMAG (2μM 17-DMAG); Vel (100nM Velcade); Ever (10μM Everolimus). Data were compared in two ways–First, using two-way ANOVA with Tukey’s multiple comparisons test to compare effects of each drug within each sample (HANL or HANLCD63). In HANLCD63 samples bafilomycin was significantly more effective in increasing EV release than control (p = 0.01) and CQ (p = 0.0002). Second, we also compared individual drug effects between HANL and HANLCD63 samples using multiple t-tests. This revealed that bafilomycin and latrunculin A significantly increases EV release in HANLCD63 (p values were 1.77e-8 for Baf and 6.37e-4 for LatA).

Existing methods of bulk EV isolation and analysis create a bottleneck for efforts to advance understanding of EV biogenesis by screening compounds that modulate specific cellular machinery. To begin to assess involvement of different cellular machineries in release of CD63-containing EVs, we treated cells expressing HANL and HANLCD63 with various compounds for 5 hrs. Conditioned media was collected at the end of treatment and Nluc activity was measured (Fig 5). Note that this screening paradigm is most suitable for identifying compounds that stimulate EV release; longer EV collection times and/or cells that release high levels of EVs is likely to be needed to uncover inhibitors of EV biogenesis.

The following five groups of compounds were tested:

1. Ion balance perturbants: oubain to inhibit sodium-potassium ATPase; amiloride to block sodium channels; thapsigargin to inhibit the ER calcium channel, SERCA; MLSA1 to activate the endosomal calcium channel, TRPML1.
2. Lysosome perturbants: bafilomycin A1 to inhibit the V-ATPase proton pump; chloroquine as a lysosomotropic alkalizing agent.
3. Cytoskeleton perturbants: nocodazole to depolymerize microtubules; latrunculin A to block actin polymerization; jasplakinolide to induce actin polymerization.
4. Lipid perturbants: wortmannin to inhibit PI3 kinase; apilimod to inhibit PI3P-5-kinase; amiodarone to accumulate LBPA in endosomes [45], methyl β-cyclodextrin to reduce cellular cholesterol; U18666A to inhibit lysosomal cholesterol export; 1-aminodecylidene bis-phosphonic acid (ABPA) to inhibit acid sphingomyelinase; GW4869 to inhibit neutral sphingomyelinase.
5. Other perturbants: ML240 to inhibit VCP/p97 AAA ATPase; 17-DMAG to inhibit Hsp90; bortezomib to inhibit the proteasome; and Everolimus to inhibit mTORC1.

Fig 5 shows the effect of these compounds on extracellular Nluc released from cells expressing HANL or HANLCD63. None of the compounds tested had a significant effect on extracellular Nluc activity in HANL samples (Fig 5), showing that as tested they did not compromise cell integrity. While most of the compounds tested also did not change extracellular Nluc released by cells expressing HANLCD63, a few significantly increased it indicating an enhancement of EV biogenesis and/or release.

Most notable was the large increase in release of CD63-containing EVs triggered by treating cells with bafilomycin, which we explore further below. Compounds that modulate Ca^2+^ homeostasis also increased HANLCD63 release. TRPML1/MCOLN, a Ca^2+^ channel on the endolysosomal membrane, has been implicated in control and release of endolysosomal Ca^2+^ and connected to exosome release [46]. Treating cells with MLSA1, an activator of TRPML1, doubled HANLCD63-marked EV release, consistent with these earlier reports. Treating cells with thapsigargin, which releases ER Ca^2+^ by inhibiting the sarco/endoplasmic reticulum Ca^2+^ ATPase (SERCA), also increased EV release, similar to previous observations [47]. These results suggest that increases in Ca^2+^ from multiple sources can enhance EV release.

We also observed that compounds affecting the actin cytoskeleton, but not microtubules, increased EV release. Both stabilizing actin filaments with jasplakinolide and inhibiting actin polymerization with latrunculin A increased EV release in our screen, potentially by relieving restrictions on exocytosis or enhancing blebbing of the plasma membrane. While we expected that transport of endosomes/MVBs along microtubules might be important for exocytosis and EV release, the lack of effect of nocodazole did not support such a role over the time course measured. Further studies will be required to determine the exact role of the cytoskeleton in EV biogenesis and/or release.

None of the lipid perturbants tested caused a significant change in CD63-EV release. Specifically, depleting cellular cholesterol with methyl-β-cyclodextrin or increasing it with U18666A had no effect on EV release. U18666A has previously been reported to have both stimulatory [48] as well as inhibitory roles in EV biogenesis [49]. Another lipid perturbant, apilimod, has been shown to cause a 50% increase in EV release over 20h of treatment [50]. Small stimulatory effects of this magnitude may not be obvious over the time course of our screen. Inhibitory effects of drugs such as GW4869 previously shown to act over 16h of treatment [51] were also not obvious in our screen. One possible explanation is that they would not be expected to inhibit exocytosis of existing MVBs. It is also possible that overexpressing HANLCD63 may diminish the sensitivity of EV biogenesis machinery to inhibitory drugs. Inhibitors of VCP/p97, Hsp90 or the proteasome also did not have significant effects at the concentrations tested. Finally, everolimus, a rapalog inhibitor of mTORC1 leading to hyperactivation of AKT signaling reproducibly enhanced EV release (Fig 5).

As above, we saw the most striking increase in EV release following treatment with bafilomycin, which inhibits the V-ATPase proton pump and thereby allows the lumenal pH of lysosomes to rise over seconds to minutes [52]. The observed increase in CD63-EV release is consistent with previous reports of bafilomycin enhancing various types of EV release [47,53,54], effects that have been attributed to the pH neutralizing effects of inhibiting the V-ATPase. Curiously, however, we found that treating cells with chloroquine, which rapidly neutralizes lumenal pH as a weak base, did not have any effect on HANLCD63 release. We set out to explore the basis for this striking dichotomy.

**Fig 6:**
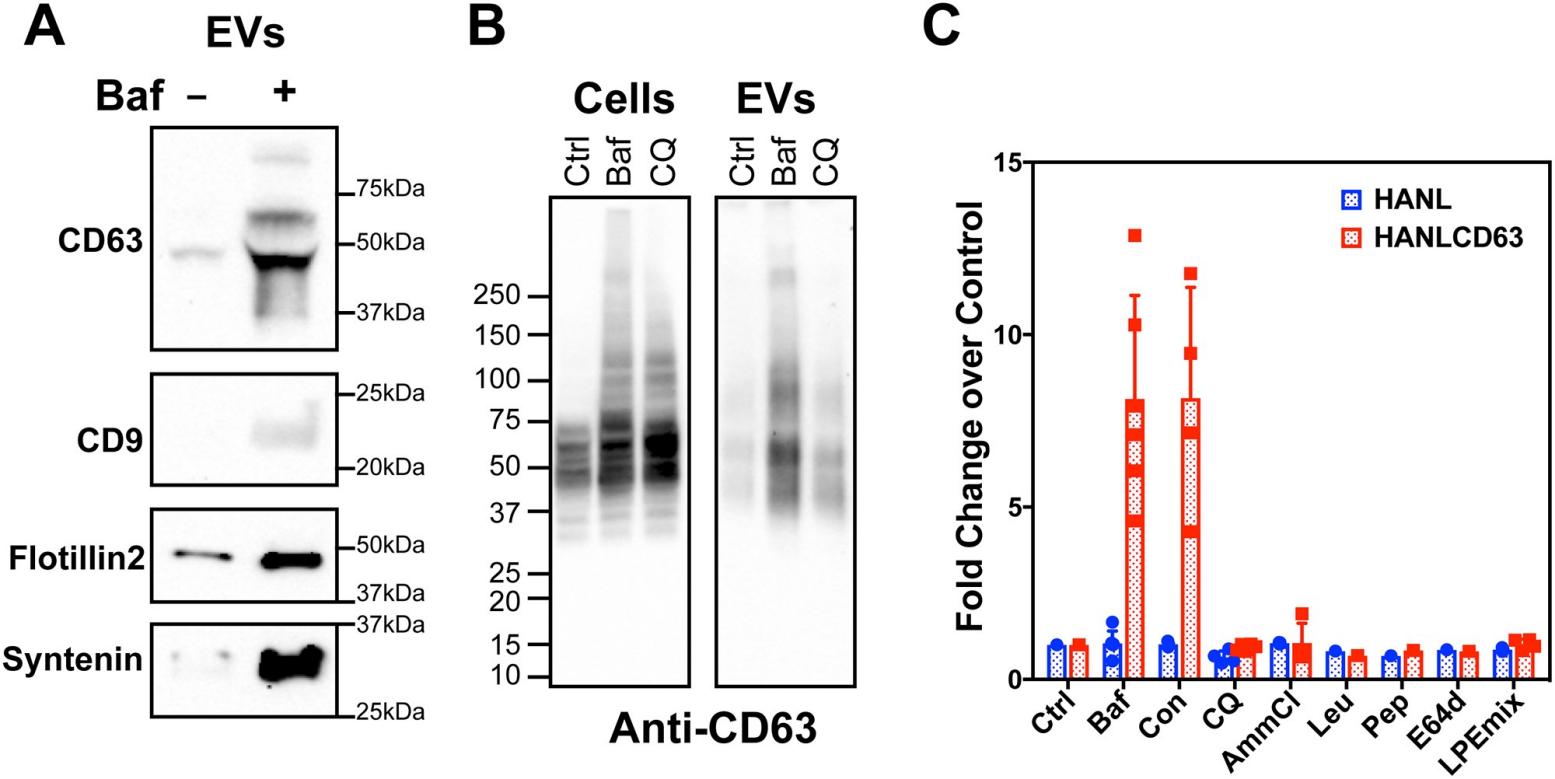
Effects of lysosomal perturbants on EV release. (A) Analysis of EVs collected from naïve HEK293 cells treated with DMSO vehicle (-) or 200nM bafilomycin (+). PEG pellets from conditioned media collected over 5 hours were immunoblotted for CD63, CD9, flotillin 2 and syntenin as markers of EVs. (B) HEK293 cells were treated with vehicle (Ctrl), 200nM bafilomycin (Baf) or 50μM chloroquine (CQ). Cell lysates (Cells) or PEG pellets of conditioned media (EVs) were immunoblotted for CD63. (C) TRex293 cells expressing HANL or HANLCD63 were treated with indicated compounds to inhibit various aspects of lysosome function. Nluc activity was measured in conditioned media and expressed as fold change over vehicle treated (Ctrl) samples. Compounds used include–(i) V-ATPase inhibitors: Baf (200nM Bafilomycin); Con (100nM Concanamycin); (ii) weak bases: CQ (50μM Chloroquine); AmmCl (2mM Ammonium Chloride); (iii) protease inhibitors: Leu (2.5μM Leupeptin); Pep (15μM Pepstatin); E64d (20μM E64d); LPE mix (2.5μM Leupeptin, 15μM Pepstatin, 20μM E64d).

First, we asked whether bafilomycin enhances EV release from naïve, untransfected, HEK293 cells using traditional EV isolation and immunoblotting techniques. After treating HEK293 cells with vehicle or bafilomycin for 5 hrs, we examined the accumulation of marker proteins including CD63, CD9, flotillin and syntenin in PEG-concentrated EVs. Bafilomycin treatment robustly increased release of all of these EV-associated proteins, consistent with a broad based increase in EVs (Fig 6A). These results demonstrate that increased EV release following treatment with bafilomycin is a general phenomenon and not specific to cells overexpressing HANLCD63.

Next, to understand whether the difference in effects of the V-ATPase inhibitor and an alkalizing agent on EV release might be due to differing effects on the total amount of cellular CD63, we compared CD63 levels by immunoblotting cell lysates and PEG-concentrated EVs after treatment with bafilomycin or chloroquine (Fig 6B and S3 Fig). Cell-associated CD63 increased comparably after 5 hours in either bafilomycin or chloroquine, suggesting that both interfere with normal lysosomal activity. However, EVs isolated from these same cells showed that treatment with bafilomycin increased EV-associated CD63 far more dramatically than did treatment with chloroquine.

In order to test whether the enhanced release caused by bafilomycin is specific to EV secretion, we examined its effect on the secretion of proteins that pass through the classical secretory pathway and do not intersect EV biogenesis pathways. Specifically, we examined secreted nanoluciferase (SecNL), a model protein for classical secretion developed by fusing IL6 secretion signal to nanoluciferase (S4 Fig). Extracellular Nluc activity was measured in cells expressing HANLCD63 or SecNL during 2h after addition of bafilomycin. The extracellular:intracellular ratio (fractional release) for SecNL increased to >600% within 2h under untreated conditions and was greatly inhibited by bafilomycin. Extracellular release of HANLCD63 was enhanced by bafilomycin as before. Bafilomycin has been previously observed to interfere with classical secretion [55,56]. These results together with our results showing no effect of bafilomycin on release of cytoplasmic HANL demonstrate that the stimulatory effect of bafilomycin is specific for EV protein release and not attributable to non-specific release of cellular proteins resulting from dying cells.

Since MVBs either fuse with lysosomes for content degradation or with the plasma membrane to release ILVs as exosomes, we posited that blocking lysosomal function with other compounds would shed further light on trigger(s) responsible for enhanced EV release. Using HANLCD63 expressing TRex293 cells we first tested the effect of another inhibitor of V-ATPase function, concanamycin, as well as the weak base ammonium chloride [57,58]. Concanamycin stimulated EV release to levels comparable to that seen with bafilomycin, while ammonium chloride again had no effect on EV release (Fig 6C). To test whether accumulation of un-degraded proteins within lysosomes might promote HANLCD63 release, we treated cells with lysosomal protease inhibitors (leupeptin, pepstatin, E64d) and measured extracellular Nluc. These inhibitors, either singly or in combination, also had no effect on HANLCD63 release suggesting that accumulation of undegraded proteins in endolysosomes is not a strong trigger for EV release (Fig 6C). To understand whether the stimulatory effect of V-ATPase inhibitors depends on changing organelle pH, we treated cells with bafilomycin together with ammonium chloride or chloroquine. Stimulated release of HANLCD63 by V-ATPase inhibition was unaffected by alkalizing agents (S5 Fig), suggesting that inhibiting V-ATPase alone was necessary and sufficient to promote enhanced release of HANLCD63.

## Discussion

We report development of a facile cell-based assay system for sensitively quantifying release of CD63-containing EVs in small volumes of conditioned culture media as a distinct phenomenon from non-specific cell lysis. To do this, we fused a small ATP-independent luciferase to the endolysosomal tetraspanin CD63 as a reporter for EVs. In addition, luciferase localized to the cytoplasm was used as a reporter for non-specific release cytoplasmic due to damage and injury to cells. This enables direct determination of EV marker release following different cell treatments. Using these markers, we established that different cultured cell lines differ in EV production. We also observed that EVs are constitutively and continuously released over a several hour time period, again distinct from cytosolic leakage. This system enabled a first pass broad-spectrum screen using compounds with known mechanistic targets, paving the way for future delineation of mechanisms of EV biogenesis. An important finding from this screen was the importance of V-ATPase activity in regulating EV release.

Our choice of luciferase was nanoluciferase (Nluc) because of its small size (19kDa), high stability and bright glow-type luminescence that is stable for >30 min. Nluc luminescence is ATP-independent and ~150 times brighter than firefly or *Renilla* luciferase [59]. Further, Nluc has no disulfide binds and is therefore well suited for expression in the cytoplasm and on the cytoplasmic extensions of transmembrane proteins such as CD63; this is distinct from another small and bright luciferase derived from *Gaussia* which has many disulfide bonds and is therefore better suited for expression on lumenal extensions of transmembrane proteins [60]. Luciferase-based tracking of exosomes and other EVs has been previously explored. Danzer *et al*., used a split *Gaussia* luciferase system to investigate the packaging and release specifically of aggregated αSynuclein as cargo within exosomes in cultured cells [61]; the required aggregation step limited quantitative interpretation of EV release. Others have used membrane localized versions of *Gaussia* luciferase to monitor EVs *in vivo* [62,63]. While this manuscript was under preparation, another report of Nluc fused to the C-terminal end of CD63 confirms the general utility of this tracer for tracking CD63-labelled membranes and EVs *in vitro* and *in vivo* [64]. However, C-terminal fusion of tracers to CD63 may perturb function of the GYXXΦ sorting signal present at the C-terminus of CD63 [42,65,66].

As proof-of concept for using this system in pharmacological screens, we tested the effect of treating Nluc marker expressing cells with compounds known to perturb ionic homeostasis, lysosomal function, cytoskeletal structure and dynamics, lipid metabolism and proteostasis. Our screening paradigm with a short EV collection time was better suited to identifying compounds that enhance EV release. Results from this screen demonstrate that inhibitors of the V-ATPase, bafilomycin and concanamycin, were profound stimulators of EV release demonstrating the utility of this facile assay system in drug and genetic screens. However, with short EV collection times we were unable to validate the effects of the previously described inhibitor of EV biogenesis GW4869. This was potentially because the incubation period of 5h is shorter than that (16h) reported in literature [51] and needed to observe an effect or because overexpression of HANLCD63 may enhance EV biogenesis and obscure the effect of inhibitory drugs. Increases in the duration of drug treatment and EV collection may make the assay more suitable for screening for inhibitors of EV biogenesis.

Our findings of bafilomycin-stimulated EV release are in agreement with earlier observations showing increased secretion of lysosomal enzymes upon treatment with bafilomycin [53]. Bafilomycin-mediated stimulation of EV release was also observed in a recent paper [54], wherein it was also shown that in HeLa cells, exosomes are retained on the plasma membrane by the action of tetherin. These authors also demonstrated that treatment of cells with bafilomycin interrupts cholesterol trafficking leading to cholesterol accumulation in endosomes and exosomes. In our study, we found that compounds that increase or decrease cholesterol did not alter EV release suggesting that accumulation of endosomal cholesterol was not likely to be the trigger for increased EV release. In K562 cells, small increases were observed in exosome release after treatment with either bafilomycin or chloroquine [47]. Importantly, in our HEK293-based system, unlike bafilomycin treatment, EV release could not be increased either by alkalizing of endosomal lumen or by inhibiting lysosomal proteases. This suggests that neither the lumenal pH of endosomes nor the lysosomal degradative capacity was responsible for increased exocytosis. An *in vivo* study in *C*. *elegans* revealed that the V-ATPase mediated apical secretion of exosomes containing hedgehog-related proteins [67].

How might V-ATPase inhibition by bafilomycin or concanamycin stimulate EV release? EV biogenesis can be influenced by perturbations at various steps including generation of intralumenal vesicles, endosomal trafficking of CD63, motility of endosomes as well as exocytosis. It is possible that the V-ATPase complex itself might somehow be responsible for increased exocytosis. Bafilomycin and concanamycin bind to V_0_-ATPase complex presumably at the interface of the a- and c-subunits that sits in the lipid bilayer [68–73]. Binding of these inhibitors is thought to interfere with the helical swiveling required for proton translocation [74]. It is thought that association and dissociation of V_1_-ATPase complex with the V_0_ complex is a key regulatory step for many cellular pathways [74] including exocytosis of synaptic vesicles [75]. The V_0_-V_1_ ATPase complex has been shown to recruit key signaling machinery such as the mTOR kinase that sense endolysosomal function and generate an appropriate response to regulate protein synthesis and cholesterol transport (reviewed in [76,77]). Kozik et al, found an important role for V-ATPase in clathrin-coated vesicle formation [78]. Johnson et al, found that lower V-ATPase activity coincided with localization of endolysosomes to the cell periphery [52], which could increase the probability of exocytosis and EV release. Merkulova et al, mapped several protein-protein interactions with the B1 subunit of the cytoplasmic V_1_ ATPase complex and found that some (eg, rabconnectin 3) were involved in Rab3-mediated exocytosis [79]. V-ATPase was found to be required for Rab27B-dependent invasive growth and metastasis in breast cancer [80] suggesting that V-ATPase and the Rab GTPase implicated in exosome biogenesis were functionally connected. Clear understanding of whether bafilomycin increases or decreases the abundance of the V_0_-V_1_ holoenzyme and its role in the association of exocytic machinery will illuminate its mode of action in exocytosis.

Because MVBs can target lumenal cargo proteins for either degradation by fusion with lysosomes or extracellular release by fusion with plasma membrane, it is reasonable to hypothesize that the V-ATPase may contribute to general proteostasis by its ability to sense endosomal/lysosomal function in order to shunt undegraded potentially damaging proteins out of the cell by exocytosis. In support of this idea, it has been observed that in *C*. *elegans* neurons stress resulted in the expulsion of a bolus of protein aggregates by exocytosis [81]. The autophagy protein, Atg5, was shown to dissociate the V1E subunit from the V_0_-V_1_ ATPase complex to promote exosome production [82]. These observations suggest that reduced V-ATPase function due to accumulation of protein aggregates in endosomes/lysosomes may lead to extracellular release of potentially pathogenic protein aggregates which in turn could spread disease pathology in the brain. Further work is needed to delineate the underlying mechanisms by which V-ATPase facilitates EV release.

## Conclusions

In this study, we have developed a novel model system for quantitatively assaying small amounts of CD63-containing EVs. We have demonstrated the utility of such a model system in screening for compounds affecting pathways important in cargo-selection, packaging and exocytosis events leading to EV release. Using this novel model system, we have identified the V-ATPase as an important regulator of EV secretion. Given the bright luminescence from Nluc, we predict that the construct presented in this paper may also be successfully used *in vivo* in surface as well as deep tissues as previously demonstrated [83].

## Supporting information

S1 Table List of antibodies used in this study. (DOCX)

S1 Fig Expression and endosomal localization of HANLCD63 in U2OS cells. (A) Quantitation of the CD63 blot in Fig 1B. Six independent bands were identified in Tet-induced samples (red bars, +++ and green bars, +) whereas five bands were identified in uninduced samples (blue bars, -). An image of the gel with the respective bads is shown as an inset. (B) U2OS (tet-inducible) cells stably transfected with HANLCD63 immunoblotted for CD63 with or without induction of exppression with tetracyclin. (C) Immunofluorescence of stably transfected cells with HA tag (green) and Lamp1 (red) shows endosomal localization of HANLCD63 in Lamp1-positive late endosomes.
(PDF)

S2 Fig Triton X-100 does not affect nanoluciferase activity in EVs. EVs were isolated from HANL and HANLCD63 expressing TRex293 cells by ultracentrifugation at 100000g for 90 minutes at 4C. The EV pellets were resuspended in PBS containing no detergent (-Tx100) or with 0.1%Triton X-100 (+Tx100) and the NLuc luminescence was measured. No difference was obvious between -Tx100 and +Tx100 samples demonstrating that addition of Tx100 to NLuc samples does not affect luminescence.
(PDF)

S3 Fig Quantitation of the blots in Fig 6B. Band intensities within equal sized boxes in each lane of the blots for cell lysates and EVs was normalized to the intensity in the respective control (Ctrl) sample.
(PDF)

S4 Fig Bafilomycin does not stimulate release of proteins secreted through classical secretion pathway. Extracellular Nluc activity was measured in HANLCD63 and SecNL for 2h under control (Ctrl; blue lines) or after addition of bafilomycin (Baf; red lines). While extracellular release of HANLCD63 was enhanced by bafilomycin, secretion of SecNL was greatly inhibited.
(PDF)

S5 Fig Ammonium chloride does not affect bafilomycin-stimulated EV secretion. NLuc luminescence was measured in conditioned culture media of HANL and HANLCD63 cells treated without (-Baf) or with 200nM bafilomycin (+Baf) and were either not co-treated (-AmmCl) or co-treated with 10mM ammonium chloride (+AmmCl) as an alkalizing agent. No difference was observed in +Baf samples with or without ammonium chloride cotreatment. This result shows that alkalizing agents do not influence increased EV release due to V-ATPase inhibitors.
(PDF)

## References

[1] Tkach M, Théry C (2016) Communication by Extracellular Vesicles: Where We Are and Where We Need to Go. Cell 164: 1226–1232. 10.1016/j.cell.2016.01.043 26967288

[2] Brown M, Johnson LA, Leone DA, Majek P, Vaahtomeri K, Senfter D et al (2018) Lymphatic exosomes promote dendritic cell migration along guidance cues. J Cell Biol 217: 2205–2221. 10.1083/jcb.201612051 29650776 PMC5987709

[3] Kamerkar S, LeBleu VS, Sugimoto H, Yang S, Ruivo CF, Melo SA et al (2017) Exosomes facilitate therapeutic targeting of oncogenic KRAS in pancreatic cancer. Nature 546: 498–503. 10.1038/nature22341 28607485 PMC5538883

[4] Sinha S, Hoshino D, Hong NH, Kirkbride KC, Grega-Larson NE, Seiki M et al (2016) Cortactin promotes exosome secretion by controlling branched actin dynamics. J Cell Biol 214: 197–213. 10.1083/jcb.201601025 27402952 PMC4949450

[5] Ekstrom EJ, Bergenfelz C, von Bulow V, Serifler F, Carlemalm E, Jonsson G et al (2014) WNT5A induces release of exosomes containing pro-angiogenic and immunosuppressive factors from malignant melanoma cells. Mol Cancer 13: 88 10.1186/1476-4598-13-88 24766647 PMC4022450

[6] Boelens MC, Wu TJ, Nabet BY, Xu B, Qiu Y, Yoon T et al (2014) Exosome transfer from stromal to breast cancer cells regulates therapy resistance pathways. Cell 159: 499–513. 10.1016/j.cell.2014.09.051 25417103 PMC4283810

[7] Peinado H, Aleckovic M, Lavotshkin S, Matei I, Costa-Silva B, Moreno-Bueno G et al (2012) Melanoma exosomes educate bone marrow progenitor cells toward a pro-metastatic phenotype through MET. Nat Med 18: 883–891. 10.1038/nm.2753 22635005 PMC3645291

[8] Luga V, Zhang L, Viloria-Petit AM, Ogunjimi AA, Inanlou MR, Chiu E et al (2012) Exosomes mediate stromal mobilization of autocrine Wnt-PCP signaling in breast cancer cell migration. Cell 151: 1542–1556. 10.1016/j.cell.2012.11.024 23260141

[9] Gomes C, Keller S, Altevogt P, Costa J (2007) Evidence for secretion of Cu,Zn superoxide dismutase via exosomes from a cell model of amyotrophic lateral sclerosis. Neurosci Lett 428: 43–46. 10.1016/j.neulet.2007.09.024 17942226

[10] Fevrier B, Vilette D, Archer F, Loew D, Faigle W, Vidal M et al (2004) Cells release prions in association with exosomes. Proc Natl Acad Sci U S A 101: 9683–9688. 10.1073/pnas.0308413101 15210972 PMC470735

[11] Perez-Gonzalez R, Gauthier SA, Kumar A, Levy E (2012) The exosome secretory pathway transports amyloid precursor protein carboxyl-terminal fragments from the cell into the brain extracellular space. J Biol Chem 287: 43108–43115. 10.1074/jbc.M112.404467 23129776 PMC3522305

[12] Rajendran L, Honsho M, Zahn TR, Keller P, Geiger KD, Verkade P et al (2006) Alzheimer’s disease beta-amyloid peptides are released in association with exosomes. Proc Natl Acad Sci U S A 103: 11172–11177. 10.1073/pnas.0603838103 16837572 PMC1544060

[13] Guix FX, Corbett GT, Cha DJ, Mustapic M, Liu W, Mengel D et al (2018) Detection of Aggregation-Competent Tau in Neuron-Derived Extracellular Vesicles. Int J Mol Sci 19: 663. 10.3390/ijms19030663 PMC5877524 29495441

[14] Wang Y, Balaji V, Kaniyappan S, Krüger L, Irsen S, Tepper K et al (2017) The release and trans-synaptic transmission of Tau via exosomes. Mol Neurodegener 12: 5 10.1186/s13024-016-0143-y 28086931 PMC5237256

[15] Saman S, Kim W, Raya M, Visnick Y, Miro S, Saman S et al (2012) Exosome-associated tau is secreted in tauopathy models and is selectively phosphorylated in cerebrospinal fluid in early Alzheimer disease. J Biol Chem 287: 3842–3849. 10.1074/jbc.M111.277061 22057275 PMC3281682

[16] Feng Z, Hensley L, McKnight KL, Hu F, Madden V, Ping L et al (2013) A pathogenic picornavirus acquires an envelope by hijacking cellular membranes. Nature 496: 367–371. 10.1038/nature12029 23542590 PMC3631468

[17] Mori Y, Koike M, Moriishi E, Kawabata A, Tang H, Oyaizu H et al (2008) Human herpesvirus-6 induces MVB formation, and virus egress occurs by an exosomal release pathway. Traffic 9: 1728–1742. 10.1111/j.1600-0854.2008.00796.x 18637904 PMC2613231

[18] Rauch S, Martin-Serrano J (2011) Multiple interactions between the ESCRT machinery and arrestin-related proteins: implications for PPXY-dependent budding. J Virol 85: 3546–3556. 10.1128/JVI.02045-10 21191027 PMC3067843

[19] Cashikar AG, Shim S, Roth R, Maldazys MR, Heuser JE, Hanson PI (2014) Structure of cellular ESCRT-III spirals and their relationship to HIV budding. Elife e02184. 10.7554/eLife.02184 PMC4073282 24878737

[20] Prescher J, Baumgärtel V, Ivanchenko S, Torrano AA, Bräuchle C, Müller B et al (2015) Super-resolution imaging of ESCRT-proteins at HIV-1 assembly sites. PLoS Pathog 11: e1004677 10.1371/journal.ppat.1004677 25710462 PMC4339578

[21] Miao Y, Li G, Zhang X, Xu H, Abraham SN (2015) A TRP Channel Senses Lysosome Neutralization by Pathogens to Trigger Their Expulsion. Cell 161: 1306–1319. 10.1016/j.cell.2015.05.009 26027738 PMC4458218

[22] Dikshit N, Bist P, Fenlon SN, Pulloor NK, Chua CE, Scidmore MA et al (2015) Intracellular Uropathogenic E. coli Exploits Host Rab35 for Iron Acquisition and Survival within Urinary Bladder Cells. PLoS Pathog 11: e1005083 10.1371/journal.ppat.1005083 26248231 PMC4527590

[23] Gonzales PA, Pisitkun T, Hoffert JD, Tchapyjnikov D, Star RA, Kleta R et al (2009) Large-scale proteomics and phosphoproteomics of urinary exosomes. J Am Soc Nephrol 20: 363–379. 10.1681/ASN.2008040406 19056867 PMC2637050

[24] Hamlett ED, Goetzl EJ, Ledreux A, Vasilevko V, Boger HA, LaRosa A et al (2017) Neuronal exosomes reveal Alzheimer’s disease biomarkers in Down syndrome. Alzheimers Dement 13: 541–549. 10.1016/j.jalz.2016.08.012 27755974 PMC5812672

[25] Kuate S, Cinatl J, Doerr HW, Uberla K (2007) Exosomal vaccines containing the S protein of the SARS coronavirus induce high levels of neutralizing antibodies. Virology 362: 26–37. 10.1016/j.virol.2006.12.011 17258782 PMC7103344

[26] Conlan RS, Pisano S, Oliveira MI, Ferrari M, Mendes Pinto I (2017) Exosomes as Reconfigurable Therapeutic Systems. Trends Mol Med 23: 636–650. 10.1016/j.molmed.2017.05.003 28648185 PMC5657340

[27] Charrin S, le Naour F, Silvie O, Milhiet PE, Boucheix C, Rubinstein E (2009) Lateral organization of membrane proteins: tetraspanins spin their web. Biochem J 420: 133–154. 10.1042/BJ20082422 19426143

[28] Pols MS, Klumperman J (2009) Trafficking and function of the tetraspanin CD63. Exp Cell Res 315: 1584–1592. 10.1016/j.yexcr.2008.09.020 18930046

[29] Zimmerman B, Kelly B, McMillan BJ, Seegar TC, Dror RO, Kruse AC et al (2016) Crystal Structure of a Full-Length Human Tetraspanin Reveals a Cholesterol-Binding Pocket. Cell 167: 1041–1051.e11. 10.1016/j.cell.2016.09.056 27881302 PMC5127602

[30] Guix FX, Sannerud R, Berditchevski F, Arranz AM, Horré K, Snellinx A et al (2017) Tetraspanin 6: a pivotal protein of the multiple vesicular body determining exosome release and lysosomal degradation of amyloid precursor protein fragments. Mol Neurodegener 12: 25 10.1186/s13024-017-0165-0 28279219 PMC5345265

[31] Hurwitz SN, Cheerathodi MR, Nkosi D, York SB, Meckes DG (2018) Tetraspanin CD63 Bridges Autophagic and Endosomal Processes To Regulate Exosomal Secretion and Intracellular Signaling of Epstein-Barr Virus LMP1. J Virol 92: e01969–17. 10.1128/JVI.01969-17 29212935 PMC5809724

[32] Mathivanan S, Simpson RJ (2009) ExoCarta: A compendium of exosomal proteins and RNA. Proteomics 9: 4997–5000. 10.1002/pmic.200900351 19810033

[33] Roucourt B, Meeussen S, Bao J, Zimmermann P, David G (2015) Heparanase activates the syndecan-syntenin-ALIX exosome pathway. Cell Res 25: 412–428. 10.1038/cr.2015.29 25732677 PMC4387558

[34] Kowal J, Arras G, Colombo M, Jouve M, Morath JP, Primdal-Bengtson B et al (2016) Proteomic comparison defines novel markers to characterize heterogeneous populations of extracellular vesicle subtypes. Proc Natl Acad Sci U S A 113: E968–77. 10.1073/pnas.1521230113 26858453 PMC4776515

[35] Russo F, Di Bella S, Nigita G, Macca V, Laganà A, Giugno R et al (2012) miRandola: extracellular circulating microRNAs database. PLoS One 7: e47786 10.1371/journal.pone.0047786 23094086 PMC3477145

[36] Shurtleff MJ, Temoche-Diaz MM, Karfilis KV, Ri S, Schekman R (2016) Y-box protein 1 is required to sort microRNAs into exosomes in cells and in a cell-free reaction. Elife 5: e19276 10.7554/eLife.19276 27559612 PMC5047747

[37] Shurtleff MJ, Yao J, Qin Y, Nottingham RM, Temoche-Diaz MM, Schekman R et al (2017) Broad role for YBX1 in defining the small noncoding RNA composition of exosomes. Proc Natl Acad Sci USA 114: E8987–E8995. 10.1073/pnas.1712108114 29073095 PMC5663387

[38] Colombo M, Raposo G, Thery C (2014) Biogenesis, secretion, and intercellular interactions of exosomes and other extracellular vesicles. Annu Rev Cell Dev Biol 30: 255–289. 10.1146/annurev-cellbio-101512-122326 25288114

[39] Tkach M, Kowal J, Théry C (2018) Why the need and how to approach the functional diversity of extracellular vesicles. Philos Trans R Soc Lond B Biol Sci 373: 20160479 10.1098/rstb.2016.0479 29158309 PMC5717434

[40] Tauro BJ, Greening DW, Mathias RA, Ji H, Mathivanan S, Scott AM et al (2012) Comparison of ultracentrifugation, density gradient separation, and immunoaffinity capture methods for isolating human colon cancer cell line LIM1863-derived exosomes. Methods 56: 293–304. 10.1016/j.ymeth.2012.01.002 22285593

[41] Hall MP, Unch J, Binkowski BF, Valley MP, Butler BL, Wood MG et al (2012) Engineered luciferase reporter from a deep sea shrimp utilizing a novel imidazopyrazinone substrate. ACS Chem Biol 7: 1848–1857. 10.1021/cb3002478 22894855 PMC3501149

[42] Rous BA, Reaves BJ, Ihrke G, Briggs JA, Gray SR, Stephens DJ et al (2002) Role of adaptor complex AP-3 in targeting wild-type and mutated CD63 to lysosomes. Mol Biol Cell 13: 1071–1082. 10.1091/mbc.01-08-0409 11907283 PMC99620

[43] Betzig E, Patterson GH, Sougrat R, Lindwasser OW, Olenych S, Bonifacino JS et al (2006) Imaging intracellular fluorescent proteins at nanometer resolution. Science 313: 1642–1645. 10.1126/science.1127344 16902090

[44] Jackson CE, Scruggs BS, Schaffer JE, Hanson PI (2017) Effects of Inhibiting VPS4 Support a General Role for ESCRTs in Extracellular Vesicle Biogenesis. Biophys J 113: 1342–1352. 10.1016/j.bpj.2017.05.032 28629620 PMC5607042

[45] Piccoli E, Nadai M, Caretta CM, Bergonzini V, Del Vecchio C, Ha HR et al (2011) Amiodarone impairs trafficking through late endosomes inducing a Niemann-Pick C-like phenotype. Biochem Pharmacol 82: 1234–1249. 10.1016/j.bcp.2011.07.090 21878321 PMC7092840

[46] Samie M, Wang X, Zhang X, Goschka A, Li X, Cheng X et al (2013) A TRP channel in the lysosome regulates large particle phagocytosis via focal exocytosis. Dev Cell 26: 511–524. 10.1016/j.devcel.2013.08.003 23993788 PMC3794471

[47] Savina A, Furlan M, Vidal M, Colombo MI (2003) Exosome release is regulated by a calcium-dependent mechanism in K562 cells. J Biol Chem 278: 20083–20090. 10.1074/jbc.M301642200 12639953

[48] Strauss K, Goebel C, Runz H, Mobius W, Weiss S, Feussner I et al (2010) Exosome secretion ameliorates lysosomal storage of cholesterol in Niemann-Pick type C disease. J Biol Chem 285: 26279–26288. 10.1074/jbc.M110.134775 20554533 PMC2924046

[49] Elgner F, Ren H, Medvedev R, Ploen D, Himmelsbach K, Boller K et al (2016) The Intracellular Cholesterol Transport Inhibitor U18666A Inhibits the Exosome-Dependent Release of Mature Hepatitis C Virus. J Virol 90: 11181–11196. 10.1128/JVI.01053-16 27707921 PMC5126375

[50] Hessvik NP, Øverbye A, Brech A, Torgersen ML, Jakobsen IS, Sandvig K et al (2016) PIKfyve inhibition increases exosome release and induces secretory autophagy. Cell Mol Life Sci 73: 4717–4737. 10.1007/s00018-016-2309-8 27438886 PMC11108566

[51] Trajkovic K, Hsu C, Chiantia S, Rajendran L, Wenzel D, Wieland F et al (2008) Ceramide triggers budding of exosome vesicles into multivesicular endosomes. Science 319: 1244–1247. 10.1126/science.1153124 18309083

[52] Johnson DE, Ostrowski P, Jaumouillé V, Grinstein S (2016) The position of lysosomes within the cell determines their luminal pH. J Cell Biol 212: 677–692. 10.1083/jcb.201507112 26975849 PMC4792074

[53] Tapper H, Sundler R (1995) Bafilomycin A1 inhibits lysosomal, phagosomal, and plasma membrane H(+)-ATPase and induces lysosomal enzyme secretion in macrophages. J Cell Physiol 163: 137–144. 10.1002/jcp.1041630116 7896890

[54] Edgar JR, Manna PT, Nishimura S, Banting G, Robinson MS (2016) Tetherin is an exosomal tether. Elife 5: e17180 10.7554/eLife.17180 27657169 PMC5033606

[55] Davis EC, Mecham RP (1998) Intracellular trafficking of tropoelastin. Matrix Biol 17: 245–254. 9749941 10.1016/s0945-053x(98)90078-6

[56] Taupenot L, Harper KL, O’Connor DT (2005) Role of H+-ATPase-mediated acidification in sorting and release of the regulated secretory protein chromogranin A: evidence for a vesiculogenic function. J Biol Chem 280: 3885–3897. 10.1074/jbc.M408197200 15542860

[57] Bowman EJ, Siebers A, Altendorf K (1988) Bafilomycins: a class of inhibitors of membrane ATPases from microorganisms, animal cells, and plant cells. Proc Natl Acad Sci U S A 85: 7972–7976. 10.1073/pnas.85.21.7972 2973058 PMC282335

[58] Dröse S, Altendorf K (1997) Bafilomycins and concanamycins as inhibitors of V-ATPases and P-ATPases. J Exp Biol 200: 1–8. 9023991 10.1242/jeb.200.1.1

[59] England CG, Ehlerding EB, Cai W (2016) NanoLuc: A Small Luciferase Is Brightening Up the Field of Bioluminescence. Bioconjug Chem 27: 1175–1187. 10.1021/acs.bioconjchem.6b00112 27045664 PMC4871753

[60] Tannous BA, Kim DE, Fernandez JL, Weissleder R, Breakefield XO (2005) Codon-optimized Gaussia luciferase cDNA for mammalian gene expression in culture and in vivo. Mol Ther 11: 435–443. 10.1016/j.ymthe.2004.10.016 15727940

[61] Danzer KM, Kranich LR, Ruf WP, Cagsal-Getkin O, Winslow AR, Zhu L et al (2012) Exosomal cell-to-cell transmission of alpha synuclein oligomers. Mol Neurodegener 7: 42 10.1186/1750-1326-7-42 22920859 PMC3483256

[62] Takahashi Y, Nishikawa M, Shinotsuka H, Matsui Y, Ohara S, Imai T et al (2013) Visualization and in vivo tracking of the exosomes of murine melanoma B16-BL6 cells in mice after intravenous injection. J Biotechnol 165: 77–84. 10.1016/j.jbiotec.2013.03.013 23562828

[63] Lai CP, Mardini O, Ericsson M, Prabhakar S, Maguire CA, Chen JW et al (2014) Dynamic biodistribution of extracellular vesicles in vivo using a multimodal imaging reporter. ACS Nano 8: 483–494. 10.1021/nn404945r 24383518 PMC3934350

[64] Hikita T, Miyata M, Watanabe R, Oneyama C (2018) Sensitive and rapid quantification of exosomes by fusing luciferase to exosome marker proteins. Sci Rep 8: 14035 10.1038/s41598-018-32535-7 30232365 PMC6145919

[65] Ihrke G, Kyttala A, Russell MR, Rous BA, Luzio JP (2004) Differential use of two AP-3-mediated pathways by lysosomal membrane proteins. Traffic 5: 946–962. 10.1111/j.1600-0854.2004.00236.x 15522097

[66] Latysheva N, Muratov G, Rajesh S, Padgett M, Hotchin NA, Overduin M et al (2006) Syntenin-1 is a new component of tetraspanin-enriched microdomains: mechanisms and consequences of the interaction of syntenin-1 with CD63. Mol Cell Biol 26: 7707–7718. 10.1128/MCB.00849-06 16908530 PMC1636879

[67] Liégeois S, Benedetto A, Garnier JM, Schwab Y, Labouesse M (2006) The V0-ATPase mediates apical secretion of exosomes containing Hedgehog-related proteins in Caenorhabditis elegans. J Cell Biol 173: 949–961. 10.1083/jcb.200511072 16785323 PMC2063919

[68] Zhang J, Feng Y, Forgac M (1994) Proton conduction and bafilomycin binding by the V0 domain of the coated vesicle V-ATPase. J Biol Chem 269: 23518–23523. 8089118

[69] Peng SB, Li X, Crider BP, Zhou Z, Andersen P, Tsai SJ et al (1999) Identification and reconstitution of an isoform of the 116-kDa subunit of the vacuolar proton translocating ATPase. J Biol Chem 274: 2549–2555. 10.1074/jbc.274.4.2549 9891027

[70] Landolt-Marticorena C, Kahr WH, Zawarinski P, Correa J, Manolson MF (1999) Substrate- and inhibitor-induced conformational changes in the yeast V-ATPase provide evidence for communication between the catalytic and proton-translocating sectors. J Biol Chem 274: 26057–26064. 10.1074/jbc.274.37.26057 10473553

[71] Huss M, Ingenhorst G, König S, Gassel M, Dröse S, Zeeck A et al (2002) Concanamycin A, the specific inhibitor of V-ATPases, binds to the V(o) subunit c. J Biol Chem 277: 40544–40548. 10.1074/jbc.M207345200 12186879

[72] Bowman BJ, Bowman EJ (2002) Mutations in subunit C of the vacuolar ATPase confer resistance to bafilomycin and identify a conserved antibiotic binding site. J Biol Chem 277: 3965–3972. 10.1074/jbc.M109756200 11724795

[73] Osteresch C, Bender T, Grond S, von Zezschwitz P, Kunze B, Jansen R et al (2012) The binding site of the V-ATPase inhibitor apicularen is in the vicinity of those for bafilomycin and archazolid. J Biol Chem 287: 31866–31876. 10.1074/jbc.M112.372169 22815478 PMC3442520

[74] Cotter K, Stransky L, McGuire C, Forgac M (2015) Recent Insights into the Structure, Regulation, and Function of the V-ATPases. Trends Biochem Sci 40: 611–622. 10.1016/j.tibs.2015.08.005 26410601 PMC4589219

[75] Bodzęta A, Kahms M, Klingauf J (2017) The Presynaptic v-ATPase Reversibly Disassembles and Thereby Modulates Exocytosis but Is Not Part of the Fusion Machinery. Cell Rep 20: 1348–1359. 10.1016/j.celrep.2017.07.040 28793259

[76] Thelen AM, Zoncu R (2017) Emerging Roles for the Lysosome in Lipid Metabolism. Trends Cell Biol 27: 833–850. 10.1016/j.tcb.2017.07.006 28838620 PMC5653458

[77] Abu-Remaileh M, Wyant GA, Kim C, Laqtom NN, Abbasi M, Chan SH et al (2017) Lysosomal metabolomics reveals V-ATPase- and mTOR-dependent regulation of amino acid efflux from lysosomes. Science 358: 807–813. 10.1126/science.aan6298 29074583 PMC5704967

[78] Kozik P, Hodson NA, Sahlender DA, Simecek N, Soromani C, Wu J et al (2013) A human genome-wide screen for regulators of clathrin-coated vesicle formation reveals an unexpected role for the V-ATPase. Nat Cell Biol 15: 50–60. 10.1038/ncb2652 23263279 PMC3588604

[79] Merkulova M, Păunescu TG, Azroyan A, Marshansky V, Breton S, Brown D (2015) Mapping the H(+) (V)-ATPase interactome: identification of proteins involved in trafficking, folding, assembly and phosphorylation. Sci Rep 5: 14827 10.1038/srep14827 26442671 PMC4595830

[80] Hendrix A, Sormunen R, Westbroek W, Lambein K, Denys H, Sys G et al (2013) Vacuolar H+ ATPase expression and activity is required for Rab27B-dependent invasive growth and metastasis of breast cancer. Int J Cancer 133: 843–854. 10.1002/ijc.28079 23390068

[81] Melentijevic I, Toth ML, Arnold ML, Guasp RJ, Harinath G, Nguyen KC et al (2017) C. elegans neurons jettison protein aggregates and mitochondria under neurotoxic stress. Nature 542: 367–371. 10.1038/nature21362 28178240 PMC5336134

[82] Guo H, Chitiprolu M, Roncevic L, Javalet C, Hemming FJ, Trung MT et al (2017) Atg5 Disassociates the V 1 V 0 -ATPase to Promote Exosome Production and Tumor Metastasis Independent of Canonical Macroautophagy. Developmental Cell 43: 716–730.e7. 10.1016/j.devcel.2017.11.018 29257951

[83] Stacer AC, Nyati S, Moudgil P, Iyengar R, Luker KE, Rehemtulla A et al (2013) NanoLuc reporter for dual luciferase imaging in living animals. Mol Imaging 12: 1–13. PMC4144862 24371848

