# Supplementary material for: A cell-based assay for CD63-containing extracellular vesicles
Source: PLoS One. 2019 Jul 24;14(7):e0220007. doi: 10.1371/journal.pone.0220007 (PMC6655660; doi:10.1371/journal.pone.0220007)
Supplement: S1 Table — (DOCX) [file pone.0220007.s001.docx]

**S1 Table: List of antibodies used in this study.**

| **Primary Antibody** | **Source** | **Dilution for IF** | **Dilution for IB** |
| --- | --- | --- | --- |
| Mouse anti-HA (12CA5) | Sigma | 1:1000 | 1:5000 |
| Mouse anti-CD63 (H5C6) | DSHB | 1:1000 | 1:1000 |
| Mouse anti-Lamp1 (H4A3) | DSHB | 1:1000 | n/a |
| Rabbit anti-HA | Sigma | 1:1000 | n/a |
| Rabbit anti-Lamp1 | Sigma | 1:1000 | n/a |
| Mouse anti-CD9 | ProteinTech | n/a | 1:500 |
| Mouse anti-Flotillin2 | BD | n/a | 1:500 |
| Rabbit anti-Syntenin | ProteinTech | n/a | 1:1000 |
|  |  |  |  |
| **Secondary Antibody** | **Source** | **Dilution for IF** | **Dilution for IB** |
| Goat anti-mouse IgG-Alexa 488 | Thermo Fisher | 1:1000 | n/a |
| Goat anti-rabbit IgG-Alexa 555 | Thermo Fisher | 1:1000 | n/a |
| Goat anti-mouse IgG-HRP | Thermo Fisher | n/a | 1:10000 |
| Goat anti-rabbit IgG-HRP | Thermo Fisher | n/a | 1:10000 |
|  |  |  |  |
