# Supplementary figures and images for: A cell-based assay for CD63-containing extracellular vesicles
Source: PLoS One. 2019 Jul 24;14(7):e0220007. doi: 10.1371/journal.pone.0220007 (PMC6655660; doi:10.1371/journal.pone.0220007)

**S1 Fig**

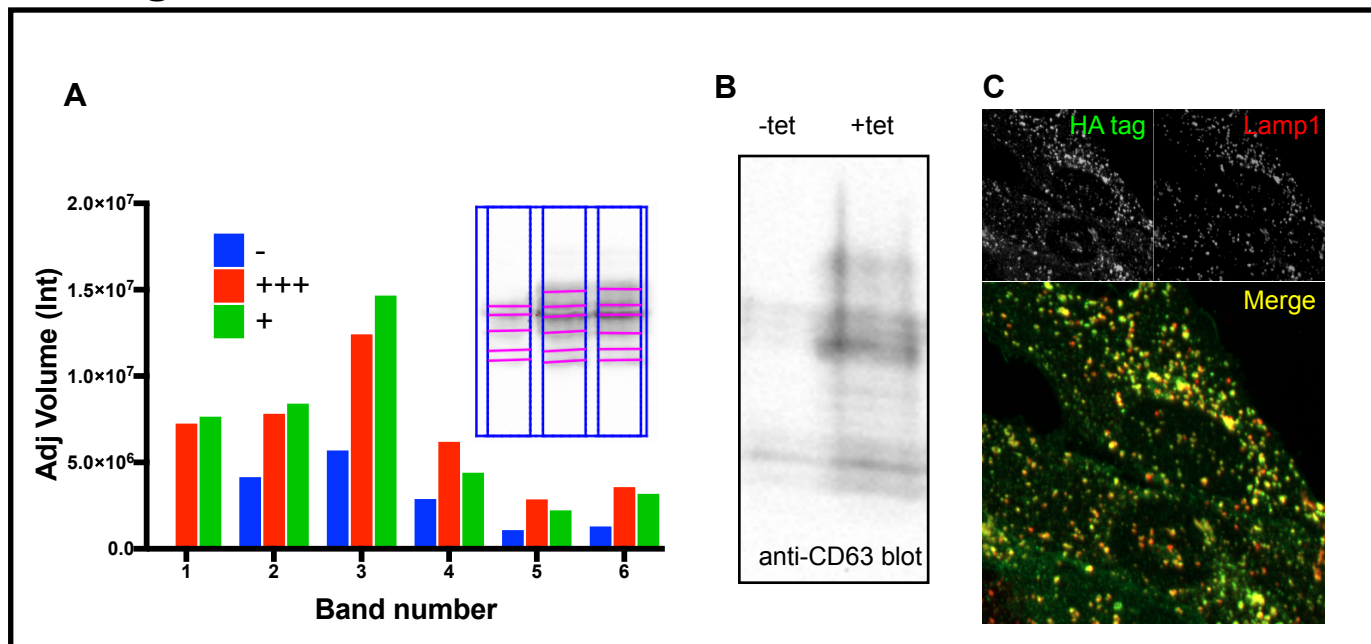

Supplement: S1 Fig — (A) Quantitation of the CD63 blot in Fig 1B. Six independent bands were identified in Tet-induced samples (red bars, +++ and green bars, +) whereas five bands were identified in uninduced samples (blue bars, -). An image of the gel with the respective bads is shown as an inset. (B) U2OS (tet-inducible) cells stably transfected with HANLCD63 immunoblotted for CD63 with or without induction of exppression with tetracyclin. (C) Immunofluorescence of stably transfected cells with HA tag (green) and Lamp1 (red) shows endosomal localization of HANLCD63 in Lamp1-positive late endosomes. (PDF) [file pone.0220007.s002.pdf]

**S2 Fig**

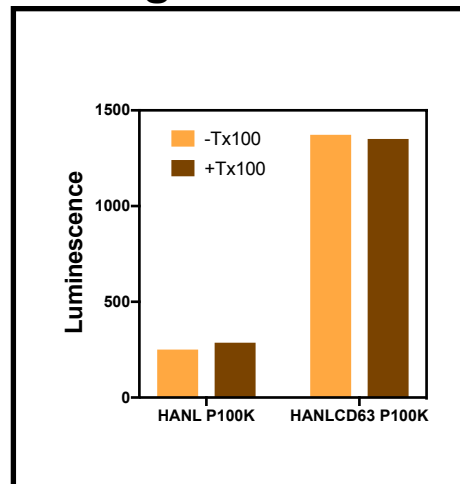

Supplement: S2 Fig — EVs were isolated from HANL and HANLCD63 expressing TRex293 cells by ultracentrifugation at 100000g for 90 minutes at 4C. The EV pellets were resuspended in PBS containing no detergent (-Tx100) or with 0.1%Triton X-100 (+Tx100) and the NLuc luminescence was measured. No difference was obvious between -Tx100 and +Tx100 samples demonstrating that addition of Tx100 to NLuc samples does not affect luminescence. (PDF) [file pone.0220007.s003.pdf]

**S3 Fig**

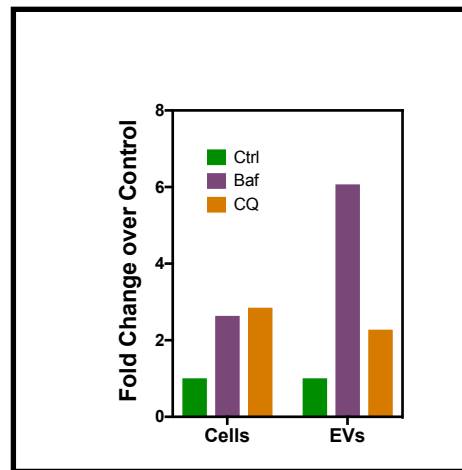

Supplement: S3 Fig — Band intensities within equal sized boxes in each lane of the blots for cell lysates and EVs was normalized to the intensity in the respective control (Ctrl) sample. (PDF) [file pone.0220007.s004.pdf]

**S4 Fig**

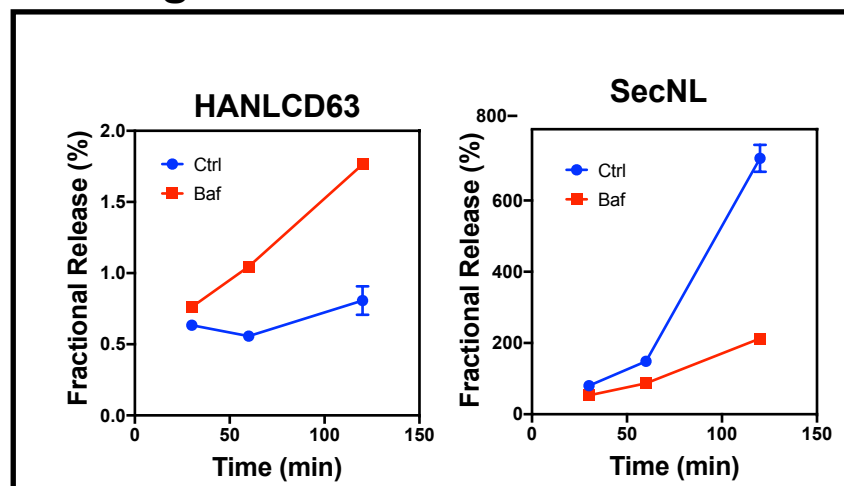

Supplement: S4 Fig — Extracellular Nluc activity was measured in HANLCD63 and SecNL for 2h under control (Ctrl; blue lines) or after addition of bafilomycin (Baf; red lines). While extracellular release of HANLCD63 was enhanced by bafilomycin, secretion of SecNL was greatly inhibited. (PDF) [file pone.0220007.s005.pdf]

**S5 Fig**

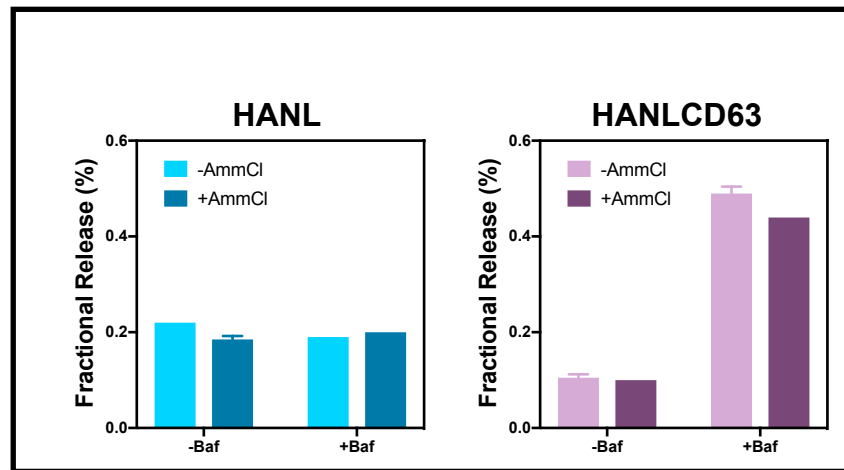

Supplement: S5 Fig — NLuc luminescence was measured in conditioned culture media of HANL and HANLCD63 cells treated without (-Baf) or with 200nM bafilomycin (+Baf) and were either not co-treated (-AmmCl) or co-treated with 10mM ammonium chloride (+AmmCl) as an alkalizing agent. No difference was observed in +Baf samples with or without ammonium chloride cotreatment. This result shows that alkalizing agents do not influence increased EV release due to V-ATPase inhibitors. (PDF) [file pone.0220007.s006.pdf]
